# Depression differed by midnight cortisol secretion, alexithymia and anxiety between diabetes types: a cross sectional comparison

**DOI:** 10.1186/s12888-017-1495-8

**Published:** 2017-09-20

**Authors:** Eva O. Melin, Maria Thunander, Mona Landin-Olsson, Magnus Hillman, Hans O. Thulesius

**Affiliations:** 1 Endocrinology and Diabetes, Department of Clinical Sciences, Lund University, Lund, Sweden; 2 Department of Research and Development, Region Kronoberg, Box 1223, SE-351 12 Växjö, Sweden; 3 Primary Care, Region Kronoberg, Växjö, Sweden; 4 Department of Internal Medicine, Central Hospital, Region Kronoberg, Växjö, Sweden; 5 Department of Endocrinology, Lund University Hospital, Lund, Sweden; 6 Diabetes Research Laboratory BMC, Lund University, Lund, Sweden; 7 Family Medicine, Department of Clinical Sciences, Lund University, Malmoe, Sweden

**Keywords:** Alexithymia, Anxiety, Depression, Cortisol, Diabetes mellitus

## Abstract

**Background:**

Increased prevalence of depression is found in both type 2 diabetes (T2D) and type 1 diabetes (T1D). Melancholia and atypical depression differ by cortisol secretion and clinical features. The aim was to compare the clinical presentation of T1D and T2D patients in relation to self-reported depression, self-reported anxiety, alexithymia, obesity, and midnight salivary cortisol (MSC).

**Methods:**

Comparative cross-sectional design. The participants were consecutively recruited from one hospital diabetes outpatient clinic: 24 T2D patients (31–59 years) and 148 T1D patients (32–59 years). Self-reported depression, anxiety and alexithymia were assessed by Hospital Anxiety and Depression scale and Toronto Alexithymia Scale-20. MSC, HbA1c, anthropometrics and data from medical records were collected. Multiple logistic regression analyses were performed.

**Results:**

Comparisons of prevalence between diabetes types showed for T2D/T1D: depression 25%/12% (*P* = 0.10); high MSC (≥9.3 nmol/L) 38%/22% (*P* = 0.13); alexithymia 25%/13% (*P* = 0.12); anxiety 38%/35% (*P* = 0.82). The prevalence of high MSC did not differ between depressed and non-depressed T2D patients (17% vs. 44%, *P* = 0.35), but differed between depressed and non-depressed T1D patients (53% vs. 18%, *P* = 0.003). The alexithymia prevalence differed between depressed and non-depressed T2D patients (67% vs.11%, *P* = 0.018), and between depressed and non-depressed T1D patients (47% vs. 11%, *P* < 0.001). The anxiety prevalence did not differ between depressed and non-depressed T2D patients (67% vs. 28%, *P* = 0.15), but differed between depressed and non-depressed T1D patients (76% vs. 30%, *P* < 0.001). The obesity prevalence (BMI ≥30 kg/m^2^) was 83% for depressed T2D patients and 6% for depressed T1D patients.

In the T2D patients, depression was associated with alexithymia (Adjusted odds ratio (AOR) 15.0). In the T1D patients, depression was associated with anxiety (AOR 11.0), foot complications (AOR 8.5), HbA1C >70 mmol/mol (AOR 6.4), and high MSC (≥9.3 nmol/L) (AOR 4.8).

**Conclusions:**

The depressed T2D patients had traits of atypical depression, without associated high MSC (≥9.3 nmol/L) and anxiety, but the association with alexithymia was strong. The depressed T1D patients had traits of melancholia with associated high MSC and anxiety. The obesity prevalence was high in depressed T2D patients and low in depressed T1D patients.

## Background

The main features of depression are dysphoria, anhedonia and lack of interest, which are accompanied by features such as weight changes, sleep disturbances, psychomotor agitation or retardation, lack of energy and/or cognitive deficits [1]. Melancholia and atypical depression are two subtypes of depression with marked differences in clinical expression [1] and in the corticotropin releasing hormone (CRH) system [2], where the changes in the CRH system are responsible for several of the clinical features [2]. In melancholia, there is an activation of the CRH system including the hypothalamic-pituitary-adrenal (HPA) axis with increased cortisol secretion, the locus coeruleus, and the sympathetic nervous system [2]. Depression in melancholia is accompanied by anxiety, a readiness to negatively charged memories, and is characterized by hyper-arousal, insomnia, loss of appetite, and weight loss. In atypical depression, there is CRH deficiency with a down-regulation of the HPA axis with decreased cortisol secretion, and a decreased sympathetic activity [2]. Atypical depression is generally not accompanied by anxiety, but is associated with a sense of emptiness, and persons with atypical depression seem to be “walled off”. The clinical picture is characterized by hypo-arousal, inactivity, hypersomnia, hyperphagia, and weight gain [2].

The prevalence of depression in diabetes is increased [3–5], in type 2 diabetes (T2D) 19–31% [3, 4], in type1 diabetes (T1D) 12–19% [4, 5], which could be compared to a prevalence of around 11% in a non-diabetic population [4]. Depression in diabetes is deleterious as it is associated with impaired glycemic control [6, 7], increased prevalence of diabetes complications, cardiovascular and all-cause mortality [8–10].

The cause of T1D is an insulin secretion deficiency due to autoimmune destruction of the pancreatic beta-cells [11]. It has been suggessted that psychological stress, mediated by excess cortisol and catecholamine secretion [12], could lead to the development of autoimmunity and the induction of T1D [13, 14]. Several biological links have been suggested between T1D and depression, one link is hyperactivity of the HPA axis which is present in both T1D and depressive states [7]. Type 2 diabetes (T2D) is characterized by insulin resistance and an inadequate compensatory insulin secretory response [11]. Obesity, which in 2009 had a prevalence of 10–11% in the Swedish population [15], is one risk factor for the development of insulin resistance and T2D [11, 16]. Depression is another risk factor for the development of T2D [3]. However, the association between T2D and depression is bidirectional, as manifest T2D also increases the risk for the development of depression [3].

As increased cortisol secretion is causative in the development of certain diabetes complications such as cognitive decline and ischemic heart disease [17–19], it is of interest to study cortisol secretion in depressed T1D and T2D patients. Even a mild increase in cortisol secretion in a non-diabetic population without clinical signs of overt hypercortisolism, is associated with an increased risk of cardiovascular events and mortality [20]. We have recently shown that high midnight salivary cortisol (MSC) (≥9.3 nmol/L) was linked to depression, physical inactivity, smoking and testing during the spring season in persons with T1D [21].

Alexithymia, a personality trait characterized by deficits in emotional awareness and expressiveness [22], has previously been linked to depression [23, 24], obesity in T1D patients [25], and to increased cardiovascular mortality [26]. We therefore consider that alexithymia might be of interest to study in the context of depression, diabetes, obesity and diabetes complications. We also hypothesize that alexithymia might be responsible for the “walled off” impression seen in patients with atypical depression. Alexithymia in depressed patients might have clinical implications. The reduced capacity of communicating feelings for persons with alexithymia, might lead to an increased risk to suffer from a depressive disorder that will not be diagnosed and thus not treated.

Given the different aetiology of T2D and T1D, the aim was to compare the clinical features of depressed patients with T2D and T1D in terms of self-reported depression, self-reported anxiety and alexithymia, MSC, and obesity. As atypical depression is characterized by hyperphagia and weight gain [2], and obesity is associated with the development of T2D [16], we hypothesize that T2D patients suffer mainly from atypical depression. As hyperactivity of the HPA axis is suggested to be a biological link between T1D and depression [7, 12], and as we previously found increased MSC in depressed T1D patients [21], we hypothesized that T1D patients mainly suffer from depression with melancholic features. To be able to differentiate between these two depression types can have clinical implications.

## Methods

### Participants and setting

**Fig. 1:**
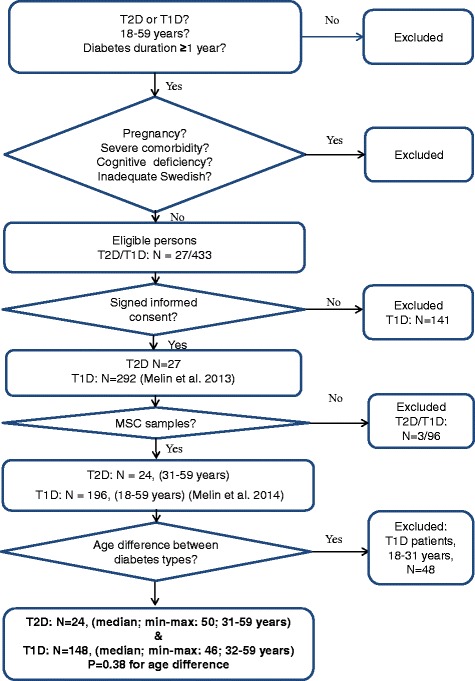
Description of the enrollment process, inclusion and exclusion criteria

This report has a comparative cross sectional design and is one of four studies [6, 21, 25], performed at baseline of a randomized controlled trial (RCT) for patients with diabetes, impaired glycemic control and psychological symptoms [27]. The participants were consecutively enrolled during a period of 9 months from March to December 2009 by specialist diabetes physicians or specialist nurses from the diabetes outpatient clinic of the Central Hospital in Växjö, Sweden. The patients consulted both physicians and nurses once a year, optionally 6 months in between. The enrolment process, inclusion and exclusion criteria are illustrated in Fig. 1. Exclusion criteria were systemic corticosteroid treatment; pregnancy; severe somatic comorbidities (cancer, hepatic failure); severe diabetes complications (end-stage renal disease, stroke with cognitive deficiency); severe mental disorders (psychotic disorder, bipolar disorder, severe personality disorder, severe substance abuse, mental retardation); visual impairment to such a degree that reading the questionnaires was impossible; or inadequate knowledge of Swedish. T1D patients younger than 32 years were excluded as we have previously shown that cortisol secretion is increasing by older age [21]. Finally, 24 T2D patients (31–59 years) and 148 T1D patients (32–59 years), who did not differ by age (*P* = 0.38), were included (Fig. 1). The T1D patients that delivered MSC samples were compared to the T1D patients of the same age that did not deliver MSC samples. Depression subtype was determined by the links between self-reported depression and self-reported anxiety and alexithymia, high MSC and general obesity. Data collection was terminated in January 2010.The study was approved by the Regional Ethical Review Board of Linköping University, Linköping (Registration no. M120–07, T89–08). All participants provided written informed consent.

### Measures

#### Data collected from the Swedish National Diabetes Register (S-NDR) and from medical records

Diabetes related data were gathered from the Swedish National Diabetes Register and from computerized medical records. Information regarding somatic and psychiatric comorbidities, and medication was collected from medical records.

Clinical psychiatric diagnoses were made prior to recruitment, and were collected from the medical records. No clinical differentiation was performed between melancholia and atypical depression by the physicians. The DSM-IV/ICD-10 depression classification codes used in the medical records were for a single episode 296.2/F32, for recurrent episodes 296.3/F33, and for unspecified depression 311/F32.9 [1]. Clinical psychiatric diagnoses were dichotomized as having or not having a clinical psychiatric diagnosis.

Diabetes complications could potentially induce depression and were therefore included in the analyses. Cardiovascular complications were defined as ischemic heart disease, stroke or transient ischemic attack. Diabetes retinopathy was defined as having non-proliferative or proliferative retinopathy, meaning microangiopathy changes as viewed by fundus photography through a dilated pupil. Foot complications were defined as significant neuropathy, angiopathy, earlier or present diabetes foot ulcer, foot infection, foot deformity, arthropathy or amputation of the lower limb. Cardiovascular complications, diabetes retinopathy and foot complications were dichotomized as having or not having the complication [6].

Smokers were defined as having smoked any amount of tobacco during the last year. Physical inactivity was defined as moderate activities, such as 30 min of walking, less than once a week.

#### Self-report measures

The patients were asked to complete the self-report instruments before leaving the out-patient clinic the day for recruitment. Self-reported depression and anxiety were assessed by the Hospital Anxiety and Depression Scale (HADS) [4, 24, 28, 29]. The depression (HADS-D) and anxiety (HADS-A) subscales, consists of 7 statements each, with 4 response alternatives from 0 to 3. The recommended cut off level ≥ 8 points (p) was used for both subscales. When the results are presented and discussed we use the term depression for self-reported depression and the term anxiety for self-reported anxiety.

Self-reported alexithymia was assessed by the Toronto Alexithymia Scale-20 items (TAS-20) which consists of 20 statements rated from 1 to 5 [30–32]. Alexithymia was defined as TAS-20 ≥ 61 p [30, 31].

#### Saliva samples, blood samples and anthropometrics

Each patient collected one MSC sample between 23.30 and 00.30 h, after oral and written information of the procedures, using the Salivette sampling method (Salivette®, Sarstedt, Nümbrecht, Germany) [21, 33]. MSC samples which were returned within 1 week after recruitment were included in the study. The samples were analyzed at the Department of Clinical Chemistry, Lund University Hospital, Lund, Sweden. The Roche Cobas Cortisolassay®, a competitive Electrochemiluminescence immunoassay (ECLIA) was used on an Elecsys 2010 immunoanalyser system (Roche Diagnostics, Mannheim, Germany) [33]. In in a healthy population sample the 95th percentile for MSC was 8.9 nmol/L [33]. High MSC was defined as ≥9.3 nmol/L, a cut-off level suggested to distinguish Cushing’s disease from pseudo-Cushing’s syndrome, corresponding to a sensitivity of 100%, a specificity of 83%, and positive/negative predictive values 90%/100% [34].

The collection period (03/29/2009–12/31/2009) was divided into three seasons: spring (03/29/2009–05/31//2009); summer (06/01/2009–08/31/2009); autumn/early winter (09/01/2009–12/31/2009). Seasonal distribution of recruited patients did not differ between diabetes types (*P* = 0.89). The number of T2D and T1D patients recruited were 9 and 60 in spring, 5 and 34 in summer, and 10 and 54 in autumn/early winter, respectively.

The 148 T1D patients that delivered MSC were compared to the 70 T1D patients (age 32–59 years) that did not deliver MSC samples. They did not differ by diabetes duration, gender, HbA1c, clinical psychiatric diagnoses, self-reported depression, self-reported anxiety, alexithymia, antidepressant use, obesity, smoking, physical inactivity; cardiovascular complications, foot complications, or diabetes retinopathy (*P*-values were between 0.21 and >0.99).

Blood samples were collected the day for recruitment. HbA1c reflects the average glucose concentration during 8–12 weeks prior to testing, and high levels indicate impaired glycemic control [6]. High HbA1c was defined as IFCC >70 mmol/mol (DCCT >8.6%).Venous HbA1c was analyzed with high pressure liquid chromatography, HPLC - variant II, Turbo analyzer (Bio – Rad®, Hercules, CA, USA) [35].

Weight and length were measured according to standard procedures by a nurse and BMI was calculated. Obesity was defined as BMI ≥30 kg/m^2^ for both genders [15].

### Statistical analysis

Analysis of data distribution using histograms revealed that MSC, HbA1c, age, diabetes duration, and BMI were not normally distributed. Data were presented as median values (quartile (q)_1_, q_3_; range), and analyses were performed with Mann-Whitney *U* test or Kruskal-Wallis test. Fisher’s exact test (two-tailed) was used to analyze categorical data. Crude odds ratios (CORs) were calculated. Multiple logistic regression analysis (Backward: Wald) were conducted separately for the two diabetes types. Variables with *P* ≤ 0.10 were entered with self-reported depression as dependent variable. The Hosmer and Lemeshow test for goodness-of-fit and Nagelkerke R^2^ were used to evaluate each regression analysis model. Confidence intervals (CIs) of 95% were used. *P* < 0.05 was considered statistically significant. SPSS® version 18 (IBM, Chicago, Illinois, USA) was used for the statistical analyses.

## Results

In this study persons with T2D (*n* = 24; 50% men) and T1D (*n* = 148; 56% men) were compared. The T2D patients were treated with insulin (25%), oral anti diabetic drugs (33%), or combinations of both (42%). The T1D patients were treated with multiple daily insulin injections (90%), or continuous subcutaneous insulin infusions (10%). The prevalence of clinical psychiatric diagnoses did not differ between diabetes types (*P* = 0.54) (Table 1). The prevalence of clinical psychiatric diagnoses was in the T2D patients 17% (clinical depression: n = 1, alcohol addiction under control: n = 1, stress related disorders: n = 2), and in the T1D patients 14% (clinical depression: *n* = 13; clinical anxiety: n = 2; stress related disorders: *n* = 5).

**Table 1:** Baseline characteristics and comparisons between 24 persons with T2D and 148 persons with T1D

		Type 2 diabetes	Type 1 diabetes	*P-*value^a^
*N*		24	148	
Age (years)		50 (41, 57; 31–59)	46 (40, 53; 32–59)	0.38^b^
Gender	Men	12 (50)	83 (56)	0.66
	Women	12 (50)	65 (44)	
Diabetes duration (years)		11 (7, 14; 2–32)	24 (14, 33; 1–55)	< 0.001^b^
Depression (HADS-D ≥ 8 p)		6 (25)	17 (12)	0.10
Alexithymia (TAS-20 ≥ 61 p)		6 (25)	19 (13)	0.12
Anxiety (HADS-A ≥ 8 p)		9 (38)	52 (35)	0.82
Physical inactivity		4 (19)	11 (8)	0.10
Smoking		2 (9)	12 (8)	> 0.99
MSC (nmol/L) All measurements		7.8 (5.1, 12.0; 1.9–23.0)	5.2 (3.1, 7.8; 1.9–47.0)	0.006^b^
MSC (nmol/L) Spring		6.7 (3.6, 11.5; 1.9–13.0)	7.1 (5.2, 10.0; 1.9–31.0)	0.84^b^
MSC (nmol/L) Summer		7.6 (7.2, 13,5; 6.8–15.0)	4.8 (3.0, 11.0; 2.3–47.0)	0.051^b^
MSC (nmol/L) Autumn/early winter		8.1 (4.5, 16.2; 3.2–23.0)	3.2 (2.7, 5.4; 1.9–26.0)	0.001^b^
High MSC (≥ 9.3 nmol/L)		9 (38)	33 (22)	0.13
HbA1c	mmol/mol	60 (49, 75; 41–113)	63 (55, 71; 32–110)	0.78^b^
	%	7.6 (6.6, 9.0; 5.9–12.5)	7.9 (7.2, 8.6; 5.1–12.2)	
HbA1c > 70 mmol/mol (> 8.6%)		8 (33)	38 (26)	0.46
BMI (kg/m^2^) Men		31 (28, 32; 26–34)	25 (23, 28; 18–38)	< 0.001^b^
BMI (kg/m^2^) Women		31 (24, 36; 21–39)	24 (23, 27; 18–45)	0.012^b^
Obesity (BMI ≥ 30 kg/m^2^)		14 (58)	16 (11)	< 0.001
Inhaled steroids		1 (4)	13 (7)	0.70
Antidepressants		2 (8)	12 (8)	> 0.99
Clinical psychiatric diagnoses		4 (17)	20 (14)	0.54
Foot complications		5 (22)	28 (20)	0.78
Cardiovascular complications		4 (17)	7 (5)	0.049
Diabetes retinopathy		14 (58)	116 (79)	0.039

Data are *n* (%) or median (q_1_, q_3_; range)

^a^Fisher’s exact test unless otherwise specified

^b^Mann - Whitney *U* test. Missing values T2D/T1D: physical inactivity *n* = 3/4; smoking n = 2/3; foot complications *n* = 1/7; diabetes retinopathy *n* = 0/1

Baseline characteristics and differences between diabetes types are presented in Table 1. The T2D patients differed from the T1D patients by higher median MSC (*P* = 0.006), higher prevalence of obesity (*P* < 0.001) and of cardio vascular complications (*P* = 0.049), lower prevalence of diabetes retinopathy (*P* = 0.039), and shorter diabetes duration (*P* < 0.001).

For the T1D patients, the MSC levels were highest when collected in spring, intermediate in summer, and lowest when collected in autumn/early winter (*P* < 0.001). There was no seasonal variation of MSC observed in the T2D patients (*P* = 0.55). The difference in MSC secretion between the two diabetes types was very high in the autumn/winter period (*P* = 0.001).

Median MSC did not differ between users and non-users of inhaled steroids (T2D: *P* = 0.94; T1D: *P* = 0.58). There were no gender differences in high MSC prevalence (T2D: *P* > 0.99; T1D: *P* = 0.56).

Differences between depressed and non-depressed T2D patients are presented in Table 2. The depressed T2D patients compared to the non-depressed had higher prevalence of alexithymia (67% versus 11*%, P* = 0.018). In the depressed T2D patients the obesity prevalence was 83%. Self-reported depression was associated with alexithymia (AOR 15.0) in 23 T2D patients (Table 2).

**Table 2:** Comparisons between depressed and non-depressed T2D patients and factors associated with depression (self-reported)

		Type 2 diabetes						
					Depression (HADS-D ≥ 8 points)			
		Depression (HADS-D ≥ 8 p)	No depression (HADS-D < 8 p)	*P*-value^a^	COR (95% CI)	*P*-value	AOR^b^ (95% CI)	*P*-value^c^
Age (years)		51 (38, 58)	48 (41, 56)	0.77^d^	1.0 (0.9–1.1)	0.80	–	–
Diabetes duration (years)		14 (10, 20)	10 (6, 13)	0.066^d^	1.11 (0.96–1.30)	0.16	–	–
Gender	Men	2 (33)	10 (56)	0.64	2.5 (0.4–17.3)	0.35	–	–
	Women	4 (67)	8 (44)					
Alexithymia (TAS-20 ≥ 61 p)		4 (67)	2 (11)	0.018	16.0 (1.7–151)	0.016	15.0 (1.6–142)	0.018
Anxiety (HADS-A ≥ 8 p)		4 (67)	5 (28)	0.15	5.2 (0.7–37.9)	0.10	Irrelevant	>0.99
Physical inactivity		1 (20)	3 (19)	>0.99	1.1 (0.09–13.5)	0.95	–	–
Smoking		1 (20)	1 (6)	0.41	4.0 (0.2–78.8)	0.36	–	–
High MSC (≥ 9.3 nmol/L)		1 (17)	8 (44)	0.35	0.25 (0.02–2.59)	0.25	–	–
HbA1c (>70 mmol/mol (> 8.6%))		4 (67)	4 (22)	0.13	7.0 (0.9–53.2)	0.06	5.8 (0.4–83.5)	0.20
Obesity (BMI ≥30 kg/m^2^)		5 (83)	9 (50)	0.34	5.0 (0.5–51.8)	0.18	–	–
Antidepressants		1 (17)	1 (6)	0.45	3.4 (0.2–64.7)	0.42	–	–
Clinical psychiatric diagnoses		2 (40)	2 (11)	0.19	5.3 (0.5–54.0)	0.16	–	–
Foot complications		3 (50)	2 (12)	0.089	7.5 (0.9–66.1)	0.07	9.9 (0.6–157)	0.10
Cardiovascular complications		2 (33)	2 (11)	0.25	4.0 (0.4–38.0)	0.23	–	–
Diabetes retinopathy		4 (67)	10 (56)	>0.99	1.6 (0.2–11.1)	0.63	–	–

^a^Fisher’s exact test unless otherwise specified

^b^Adjusted odds ratio

^c^Multiple regression analysis (Backward: Wald): T2D: *n* = 23; Hosmer and Lemeshow test 0.875; Nagelkerke R^2^ Square 0.358

^d^Mann-Whitney *U* test

Differences between depressed and non-depressed T1D patients are presented in Table 3. The depressed T1D patients compared to the non-depressed had higher prevalence of alexithymia (47% versus 8%*, P* = < 0.001), anxiety (76% versus 30%, *P* = < 0.001), high MSC (≥ 9.3 nmol/L) (53% versus 18%, *P* = 0.003). The obesity prevalence was in the depressed T1D patients 6%. Self-reported depression was associated with anxiety (AOR 11.0), foot complications (AOR 8.5), high HbA1C (AOR 6.4), and high MSC (≥9.3 nmol/L) (AOR 4.8) in 141 T1D patients, (Table 3).

**Table 3:** Comparisons between depressed and non-depressed T1D patients and factors associated with depression (self-reported)

		Type 1 diabetes						
					Depression (HADS-D ≥ 8 points)			
		Depression (HADS-D ≥ 8 p)	No depression (HADS-D < 8 p)	*P*-value^a^	COR (95% CI)	*P*-value	AOR^b^ (95% CI)	*P*-value^c^
Age (per year)		51 (43, 53)	46 (40, 53)	0.097^d^	1.1 (0.99–1.1)	0.094	1.1 (0.98–1.19)	0.14
Diabetes duration (per year)		28 (16, 37)	24 (13, 33)	0.45	1.01 (0.97–1.06)	0.50	–	–
Gender	Men	10 (59)	73 (56)	> 0.99	0.9 (0.3–2.5)	0.81	–	–
	Women	7 (41)	58 (44)					
Alexithymia (TAS-20 ≥ 61 p)		8 (47)	11 (8)	< 0.001	9.7 (3.1–30.2)	0.022	2.7 (0.5–15.8)	0.27
Anxiety (HADS-A ≥ 8 p)		13 (76)	39 (30)	< 0.001	7.7 (2.4–25.0)	0.001	11.0 (2.5–47.7)	0.001
Physical inactivity		3 (18)	8 (6)	0.12	3.2 (0.8–13.4)	0.11	–	–
Smoking		2 (12)	10 (8)	0.63	1.6 (0.3–7.9)	0.58	–	–
High MSC (≥ 9.3 nmol/L)		9 (53)	24 (18)	0.003	5.0 (1.8–14.3)	0.003	4.8 (1.1–20.1)	0.034
HbA1c (>70 mmol/mol (> 8.6%))		10 (59)	28 (21)	0.002	5.3 (1.8–15.1)	0.002	6.4 (1.6–26.0)	0.009
Obesity (BMI ≥30 kg/m^2^)		1 (6)	15 (12)	0.70	0.5 (0.1–3.9)	0.50	–	–
Antidepressants		4 (24)	8 (6)	0.034	4.7 (1.3–17.9)	0.022	1.2 (0.2–7.3)	0.87
Clinical psychiatric diagnoses		7 (41)	13 (10)	0.002	6.4 (2.1–19.5)	0.001	–	–
Foot complications		8 (50)	20 (16)	0.004	5.2 (1.8–15.6)	0.003	8.5 (1.9–37.4)	0.004
Cardiovascular complications		3 (18)	4 (3)	0.033	6.8 (1.4–33.5)	0.018	2.6 (0.3–22.6)	0.39
Diabetes retinopathy		15 (88)	101 (78)	0.53	2.2 (0.5–10.0)	0.33	–	–

^a^Fisher’s exact test unless otherwise specified

^b^Adjusted odds ratio

^c^Multiple regression analysis (Backward: Wald): T1D: *n* = 141; Hosmer and Lemeshow test 0.571; Nagelkerke R^2^ Square 0.465

^d^Mann-Whitney *U* test

## Discussion

In this comparative study of self-reported depression and associated features in 24 patients with T2D and 148 patients with T1D, consecutively recruited from one hospital diabetes outpatient clinic, the depression prevalence was twice as high in the T2D patients as in the T1D patients. The clinical presentation of depression in the T1D and T2D patients differed by associated anxiety, alexithymia, obesity, and midnight salivary cortisol secretion. Depression in the T2D patients was associated with alexithymia, but not with anxiety or with high midnight cortisol secretion (≥ 9.3 nmol/L). The obesity prevalence was very high in the depressed T2D patients. In the T1D patients, depression was associated with anxiety, high midnight cortisol secretion, impaired glycemic control (HbA1c > 70 mmol/mol (> 8.6%)) and with foot complications. The obesity prevalence in the depressed T1D patients was low.

The findings of our research support our hypothesis that depressed T2D patients mainly suffer from features of atypical depression, and depressed T1D patients mainly suffer from features of melancholic depression. We have not found any previous study where features of melancholia and atypical depression were compared between the two diabetes types. As increased cortisol secretion is causative in the development of cognitive and cardiovascular complications, it is of utmost importance to treat depression in T1D due to the demonstrated increase in cortisol secretion [17–19]. As previously described, atypical depression is not accompanied by anxiety, but is characterized by a down regulation of the HPA axis, hypo-arousal, weight gain during depressive episodes, and a sense of emptiness [2]. Alexithymia might be the reason for both the feelings of emptiness, and the “walled off” impression on other people that are clinical features of atypical depression [2]. The low emotional awareness and the reduced capacity of communicating feelings, features of alexithymia [22], might have clinical implications. First, there is a risk that depression in persons with alexithymia will remain undiagnosed as alexithymic persons have difficulties recognizing and communicating their own feelings, thus also their depressive state. When meeting an inactive, obese patient with diabetes, inadequate glycemic control, who seems to be walled off, we suggest that the patient could be tested with a self-report instrument for depression, such as the Hospital Anxiety and Depression Scale (HADS [4, 24, 28, 29], and a self-report instrument for alexithymia, Toronto Alexithymia Scale-20 items (TAS-20) [29–32]. Second, alexithymic depressed persons might benefit from different types of psychotherapy than non-alexithymic depressed persons. Third, interventions targeting increased emotional awareness in weight reduction programs for patients with obesity and alexithymia, with or without depression, might be beneficial for both diabetes types, as we have recently showed that alexithymia was associated with obesity in T1D patients [25].

The prevalence of depression were in accordance with previous research for both diabetes types, but we did not find any significant difference between the two diabetes types [3–5]. The depression prevalence would most probably have been higher for both diabetes types if we had not excluded persons with severe substance abuse, bipolar disorder, depression with psychotic features, and severe somatic comorbidities. The depression prevalence reported in previous research depends on which method that was used for the diagnosis [3–5]. Our results would probably have differed if we had performed a diagnostic interview.

Due to well-known link between impaired glycemic control and depression [7], we suggest that a self-report instrument for the detection of depression should be used routinely for all patients with diabetes and impaired glycemic control.

Compared to the obesity prevalence in the general Swedish population, the obesity prevalence was lower for the depressed T1D patients, and more than 7 times higher for the depressed T2D patients, and more than 4 times higher for the non-depressed T2D patients [15]. The high obesity prevalence in the T2D patients is deleterious as obesity significantly contributes to cardiovascular disease [36]. The associations between depression and impaired glycemic control [7], increased cortisol secretion [7], and foot complications [3, 10], in the T1D patients are in accordance with previous literature.

There are several subjects for future research. Will cortisol secretion decrease in T1D patients recovered from depression? Do physicians fail to recognize depression in persons with alexithymia? Are the features of atypical depression, hypo-arousal, inactivity, hyperphagia and weight gain, misinterpreted clinically as just laziness or non-adherence to diabetes regiment in T2D patients? Do the two depression types benefit from different antidepressants and psychotherapeutic interventions? Cognitive behavioural therapy could be tried against emotion focused interventions [24, 27, 29, 37–39]. For depressed persons with increased cortisol secretion, stress reducing techniques such as mind-body therapies could be tried [27, 39]. We have previously targeted alexithymia with good results by intervention with the psychoeducational method “Affect School with Script analysis” (ASSA) [24]. We have initiated two randomized controlled studies where ASSA is tried against basic body awareness for patients with T1D or T2D, inadequate glycemic control and psychological symptoms [27].

It would also be of interest to perform a longitudinal study to explore whether the prevalence of alexithymia is increasing by age or diabetes duration. Hypothetically, alexithymia could be part of a general cognitive decline caused by diabetes [19, 40].

Strengths of our study are that the population was well defined. Users of systemic corticosteroid treatment, pregnant women, persons with severe somatic or psychiatric disorders, or with severe substance abuse, were excluded. We have previously shown that MSC increases by age [21]. By excluding younger T1D patients (< 32 years), we made sure the two populations were matched by age, so that age wouldn’t bias the association between MSC and depression. We explored whether patients that delivered MSC samples differed from those that did not, and we did not find any difference for any variable included in the study. We included diabetes complications in the analyses as the presence of complications could induce depression. We explored whether there were seasonal variations of MSC secretion in the T2D patients, as in the T1D patients [21], which was not the case.

The main limitation of our study was the small number of patients with T2D. It will be necessary to explore whether our findings of the very strong association between depression and alexithymia, and the absence of associations between anxiety, high MSC and depression in T2D can be confirmed in a larger population. To explore seasonal variation of MSC in a larger population of T2D patients would be of interest. A larger study of depression, alexithymia, anxiety, seasonal variation and MSC in persons with T2D is therefore planned in primary care settings [27].

Another limitation is that depression and anxiety were self-reported and not confirmed by a diagnostic interview. HADS has however recently been validated and showed good reliability and discriminant validity [28]. The conclusion of the validation was that HADS is a useful instrument for detecting anxiety and depression symptoms, both at an individual and a collective level [28]. In this study, the T1D patients with self-reported depression had a higher prevalence of clinical psychiatric diagnoses and antidepressant use than patients without self-reported depression, which indicates that the scale is relevant for detecting depressive symptoms.

A third limitation was that all the features that might differ between melancholia and atypical depression were not explored, for example patterns of sleeping disturbances [2].

## Conclusions

The clinical presentation of depression in T1D and T2D patients differed by associated anxiety, alexithymia, obesity, and midnight salivary cortisol secretion. The hypothesis that depressed patients with T2D mainly suffer from features linked to atypical depression was in this study supported by the very high prevalence of obesity, the association with alexithymia, the lack of associated anxiety, and lack of increment of midnight cortisol secretion in the depressed T2D patients. The hypothesis that depressed T1D patients mainly suffer from features linked with melancholic depression was supported by the presence of associated anxiety, increased midnight cortisol secretion, and the low prevalence of obesity in the depressed T1D patients. We have not found any previous study where different subtypes of depression were explored in T1D and T2D. Awareness of the existence of the two different depression sub types, melancholia and atypical depression, can have implications both for the ability to identify depression in patients with diabetes and for the treatment of depression.

## Abbreviations

AOR: Adjusted odds ration
BMI: Body mass index
CI: Confidence interval
COR: Crude odds ratio
CRH: Corticotropin releasing hormone
ECLIA: Electrochemiluminescence immunoassay
HPA: Hypothalamic-pituitary-adrenal
MSC: Midnight salivary cortisol
T1D: Type 1 diabetes
T2D: Type 2 diabetes.
